# Clinical benefit of improved Prehospital stroke scales to detect stroke patients with large vessel occlusions: results from a conditional probabilistic model

**DOI:** 10.1186/s12883-018-1021-8

**Published:** 2018-02-10

**Authors:** Ludwig Schlemm, Eckhard Schlemm

**Affiliations:** 1 Department of Neurology, Charité – Universitätsmedizin Berlin, Berlin, Germany; 2 Center for Stroke Research Berlin (CSB), Charité – Universitätsmedizin, Berlin, Germany; 3 Berlin Institute of Health (BIH), Berlin, Germany; 4 London School of Economics and Political Science, London, UK; 5 Universität Hamburg, Medizinische Fakultät, Hamburg, Germany; 6 Klinik und Poliklinik für Neurologie, Kopf- und Neurozentrum, Universitätsklinikum Hamburg-Eppendorf, Hamburg, Germany

**Keywords:** Ischemic stroke, Endovascular treatment, Thrombectomy, Thrombolysis, Prehospital triage, Emergency medical services

## Abstract

**Background:**

Clinical scales to detect large vessel occlusion (LVO) may help to determine the optimal transport destination for patients with suspected acute ischemic stroke (AIS). The clinical benefit associated with improved diagnostic accuracy of these scales has not been quantified.

**Methods:**

We used a previously reported conditional model to estimate the probability of good outcome (modified Rankin scale sore ≤2) for patients with AIS and unknown vessel status occurring in regions with greater proximity to a primary than to a comprehensive stroke center. Optimal rapid arterial occlusion evaluation (RACE) scale cutoff scores were calculated based on time-dependent effect-size estimates from recent randomized controlled trials. Probabilities of good outcome were compared between a triage strategy based on these cutoffs and a strategy based on a hypothetical perfect LVO detection tool with 100% diagnostic accuracy.

**Results:**

In our model, the additional benefit of a perfect LVO detection tool as compared to optimal transport-time dependent RACE cutoff scores ranges from 0 to 5%. It is largest for patients with medium stroke symptom severity (RACE score 5) and in geographic environments with longer transfer time between the primary and comprehensive stroke center.

**Conclusion:**

Based on a probabilistic conditional model, the results of our simulation indicate that more accurate prehospital clinical LVO detections scales may be associated with only modest improvements in the expected probability of good outcome for patients with suspected acute ischemic stroke and unknown vessel status.

**Electronic supplementary material:**

The online version of this article (10.1186/s12883-018-1021-8) contains supplementary material, which is available to authorized users.

## Background

Patients with acute ischemic stroke (AIS) due to large vessel occlusion (LVO) have better outcomes if they receive endovascular therapy (EVT) in addition to thrombolysis. [1] It is currently not known whether patients with suspected AIS should be transported directly to an EVT-capable comprehensive stroke center (CSC) even if that would mean bypassing a closer non-EVT-capable primary stroke center (PSC). [2] Recent results suggest that prehospital triage strategies based on stroke severity scale cutoff scores may be associated with higher probabilities of good outcome as compared to transportation of all patients to the nearest stroke center. [3] While a large number of different prehospital scales based on clinical symptoms and designed to detect patients with LVO exist, these prehospital LVO detection scales have limited sensitivity and specificity. [4] Therefore, research efforts are currently directed towards improving the accuracy of these scales. [5] However, the benefit for patients in terms of probability of good clinical outcome achievable with improved diagnostic accuracy of prehospital LVO detection scales has not been examined. In this article, we quantify the additional benefit of a hypothetical LVO detection tool with 100% accuracy over currently existing scales exemplified by the rapid arterial occlusion evaluation (RACE) scale. [6]

## Methods

Our analysis is based on a probabilistic model built to estimate the probability of good outcome (defined as modified Rankin Scale score ≤ 2) for patients with AIS and unknown vessel status as a function of stroke severity, transport times to the nearest PSC and CSC, and transfer times between PSC and CSC. Details of the model have been published previously. [3] Briefly, for any location on a two-dimensional temporo-spatial plane closer to a PSC than to a CSC (i.e., the region with uncertainty regarding the optimal transport destination), the optimal RACE cutoff score that should be used to determine the transport destination for a patient with suspected AIS was calculated based on the expected stroke severity-dependent probability of LVO among all patients with ischemic stroke [6] and published time-decay curves for the effects of thrombolysis and EVT. [7, 8] This optimal RACE cutoff score takes into account expected transport times to the PSC and CSC, the transfer time between the nearest PSC and CSC, as well as performance metrics at PSC and CSC (door-to-needle time, door-out-time, and door-to-groin time) and was shown to perform better than any fixed RACE cutoff score. Patients with a RACE score ≥ optimal cutoff score would be transported to the nearest CSC (mothership approach), while all other patients would be transported to the nearest PSC with secondary transfer to the CSC if needed (drip and ship approach). Stroke severity is assessed by the RACE scale (scores ranging from 0 to 9) with higher scores indicating more severe strokes. The RACE scale is a prospectively validated 5-item clinical scale that assesses facial palsy, upper and lower limb motor function, gaze deviation, and aphasia or agnosia (according to the side of hemiparesis). Using a fixed cutoff score of ≥5, its accuracy for the detection of LVO was found to be 0.72. [6] Our model assumes a physiological perspective focusing on reperfusion, which can occur after thrombolysis or EVT. It accounts for possible recanalization during secondary transfer and for reduced time to groin puncture at the CSC if the stroke team is notified in advance. Model parameters are displayed in Table 1. To quantify the added benefit of a hypothetical prehospital LVO detection tool with 100% accuracy, we calculated probabilities of good outcome for each point on the temporo-spatial plane as a function of stroke severity and transfer time between hospitals assuming that vessel status could be ascertained with certainty on scene. We then compared the results with probabilities of good outcome attainable with the currently available RACE scale. Simulations were performed in MATLAB. Results are presented as beanplots [9] generated in R. [10] The vertical spread of each bean represents the distribution of results across the temporo-spatial plane (i.e., different combinations of expected transport times to the nearest PSC and CSC). No ethical approval and no informed consent was required for this study.

**Table 1:** Parameters used in the model^a^

Parameter	Value, minutes	References
Onset-to-alarm	30	Zock et al. [14]
Alarm-to-scene	15	Federal Highway Research Institute Germany [15]
On-scene	30	Personal experience from the Berlin fire brigade [3]
Transfer (PSC-to-CSC)	15, 60, and 120	
Door-to-needle	CSC: 30 PSC: 30	
Door-out	30	Holodinsky et al., [16] Schlemm et al. [3]
Door-to-groin	Mothership approach: 90 Drip and ship approach: max(50, 90 – door-out – transfer)	
Groin-to-reperfusion	30	
Treatment time windows	Thrombolysis: 270; EVT (onset-to-groin): 360	American Heart Association [17, 18]

^a^EVT, endovascular therapy; CSC, comprehensive EVT-capable stroke center; PSC, non-EVT-capable primary stroke center

## Results

**Fig. 1:**
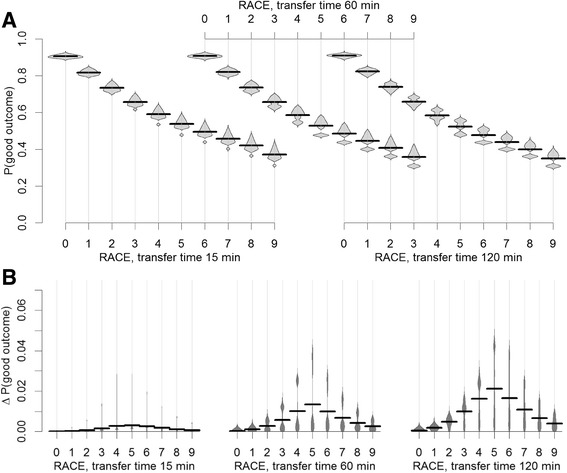
Distributions of probability of good outcome (modified Rankin scale score ≤ 2) and incremental benefit according to stroke severity and geographic setting, defined by the transfer time between primary stroke center (PSC) and comprehensive stroke center (CSC). **a** Absolute probabilities of good outcome associated with a prehospital triage strategy based on optimal rapid arterial occlusion evaluation (RACE) scale cutoff scores. **b** Point-wise differences between the probabilities of good outcome associated with a prehospital triage strategy based on a hypothetical perfect large vessel occlusion detection tool and the probabilities of good outcome associated with a prehospital triage strategy based on optimal RACE cutoff scores. The vertical spread of each bean represents the distribution of results across the temporospatial plane (i.e., different combinations of expected transport times to the nearest PSC and CSC), black horizontal lines indicate the mean

Absolute probabilities of good outcome using a prehospital triage strategy based on optimal RACE cutoff scores are displayed in Fig. 1a. Incremental benefits of a hypothetical perfect LVO detection tool range from 0 to 5%. The effect is most noticeable for medium severity strokes (RACE score 5), geographic environments with longer transfer time between hospitals, and in scenarios in which treatment time windows would allow neither thrombolysis at the CSC under the mothership approach nor secondary transfer for EVT under the drip and ship approach (Fig. 1b; Additional file 1, Panels A and B). The effect is slightly larger (maximum difference 6.7%) if the hypothetical perfect LVO detection tool is compared with a strategy based on a fixed RACE cutoff score ≥ 5 (Additional file 1, Panel B). To illustrate, in a geographic environment with a transfer time between the nearest PSC and CSC of 60 min, patients with a stroke severity corresponding to a RACE score of 4 have, on average, a ~ 58% probability of good clinical outcome if a triage strategy based on optimal variable RACE cutoff scores is used (Fig. 1a). Depending on the specific combination of expected transport times to the PSC and CSC, this probability varies between ~ 51% and ~ 64% (vertical spread of the bean in Fig. 1a). If a triage scale with 100% accuracy was available, this would increase the probability of good clinical outcome by 1 percentage point (range 0–3.1 points). In this example, the incremental benefit of a perfect LVO detection tool over a strategy based on a fixed RACE cutoff score ≥ 5 would be 1.8 points (range 0–5.6 points).

## Discussion

We quantify the clinical benefit potentially achievable with improved prehospital LVO detection scales. Depending on stroke symptom severity, the geographic environment, and the location of stroke incidence relative to the surrounding stroke centers, the probability of good outcome increases by 0–5%. The largest difference is seen for medium severity strokes (RACE score 5, because for these patients, the association between clinically observable severity and vessel status is most ambiguous (probability *p* of LVO: ~ 50%; corresponding binary Shannon entropy *-p x log2 p – (1-p) x log2 (1-p):* ~ 1.00; Additional file 2).

Besides ongoing efforts to improve clinical LVO detection scales, there has recently been a growing interest in the use of portable technologies such as ultrasound [11] and computed tomography angiography [12] for the prehospital identification of patients with LVO. In contrast to clinical scales, the latter also offers the possibility to exclude intracranial hemorrhage and to start thrombolysis on scene, potentially improving patient outcome independently of improved triage. However, these technologies require either extensive training or financial investments and have therefore not been adopted widely.

The results of our simulation suggest that further improvement of existing LVO detection scales or the development of new scales with higher accuracy would lead to only small improvements in the expected probability of good outcome for patients with unknown vessel status. This is due to a) the relatively large number of patients with mild stroke symptoms in an unselected sample who are less likely to have LVO and have a higher absolute probability of good outcome and b) the fact that the sensitivity of existing LVO detection scales is already high (85% for a RACE cutoff score ≥ 5). Availability of clinical scales with better discriminatory power would be nonetheless highly desirable. First, each falsely negative triage decision to a PSC may be associated with delayed access to EVT and permanent disability for an individual patient and should therefore be avoided. Second, from a societal perspective, a hypothetical scale with perfect accuracy would eliminate negative consequences of prehospital triage caused by low specificity and negative predictive value of currently available LVO detection scales, such as overburdening of CSCs with non-LVO infarcts, intracerebral hemorrhages and stroke mimics and deskilling and deprioritizing of PSCs due to lower patient volume. [2, 13]

The limitations of our model have been described previously. [3] Briefly, these include a) assumption of a uniform distribution of strokes in the temporo-spatial plane; b) the lack of stratification according to age due to the unavailability of joint distributions of good outcome with regards to age and treatment time; c) the derivation of model parameters from effect size estimates observed in recent randomized controlled trials, [7, 8] which might be different from those achievable in clinical practice; and d) the assumption of homogenous time-decay curves for EVT and thrombolysis across patients with potentially different stroke aetiologies. Confirmation of our findings in clinical studies is therefore needed once improved scales become available.

## Conclusions

In conclusion, our results indicate that more accurate prehospital clinical LVO detections scales may be associated with only modest improvements in the expected probability of good outcome for patients with suspected acute ischemic stroke and unknown vessel status. Development of LVO detection scales with higher specificity could help to minimize the negative effects on patient distribution and resource use associated with currently available triage scales.

## Additional files

Additional file 1: Incremental benefit of a hypothetical perfect large vessel occlusion detection scale according to treatment scenario I – IV. **Panel A**: Displayed are distributions of point-wise differences between the estimated probabilities of good outcome (modified Rankin scale score ≤2) associated with a prehospital triage strategy based on a hypothetical perfect large vessel occlusion (LVO) detection tool and the estimated probabilities of good outcome associated with a prehospital triage strategy based on optimal rapid arterial occlusion evaluation (RACE) scale cutoff scores, for different stroke severities and different transfer time settings (left to right: 15, 60, and 120 min). **Panel B**: Displayed are distributions of point-wise differences between the estimated probabilities of good outcome (modified Rankin scale score ≤2) associated with a prehospital triage strategy based on a hypothetical perfect LVO detection tool and the estimated probabilities of good outcome associated with a prehospital triage strategy based on a RACE cutoff score ≥ 5, for different stroke severities and different transfer time settings (left to right: 15, 60, and 120 min). Colors represent different treatment scenarios, with the possibility for treatment options defined by standard treatment time windows: symptom onset-tothrombolysis – 270 min; symptom onset-to-groin puncture – 360 min): I, both thrombolysis at the comprehensive stroke center (CSC) under the mothership approach and secondary transfer for endovascular therapy (EVT) under the drip and ship approach possible. II, thrombolysis at the CSC under the mothership approach not possible, secondary transfer for EVT under the drip and ship approach possible. III, thrombolysis at the CSC under the mothership approach possible, secondary transfer for EVT under the drip and ship approach not possible. IV, neither thrombolysis at the CSC under the mothership approach nor secondary transfer for EVT under the drip and ship approach possible. Horizontal lines indicate means. (TIFF 2376 kb)

Additional file 2: Relationship between the uncertainty with regards to the presence of large vessel occlusion and the incremental benefit of a hypothetical perfect large vessel occlusion detection tool over optimal rapid arterial occlusion evaluation scale cutoff scores. Uncertainty with regards to the presence of large vessel occlusion (LVO) among patients with ischemic stroke in each rapid arterial occlusion evaluation (RACE) scale score category is quantified using the Shannon entropy *-p x log2 p - (1-p) x log2 (1-p)*. Here, p denotes the probability of the presence of LVO among patients with ischemic stroke in a given RACE score category. Incremental benefits of a hypothetical perfect LVO tool correspond to average values in a geographic environment with a transfer time between PSC and CSC of 120 minutes. Labels indicate RACE score categories. (TIFF 1292 kb)

## Abbreviations

AIS: acute ischemic stroke
CSC: endovascular-therapy-capable comprehensive stroke center
EVT: endovascular therapy
LVO: large vessel occlusion
PSC: non-endovascular-therapy-capable primary stroke center
RACE: rapid arterial occlusion evaluation (scale)
