# Supplementary figures and images for: Clinical benefit of improved Prehospital stroke scales to detect stroke patients with large vessel occlusions: results from a conditional probabilistic model
Source: BMC Neurol. 2018 Feb 10;18:16. doi: 10.1186/s12883-018-1021-8 (PMC5807751; doi:10.1186/s12883-018-1021-8)

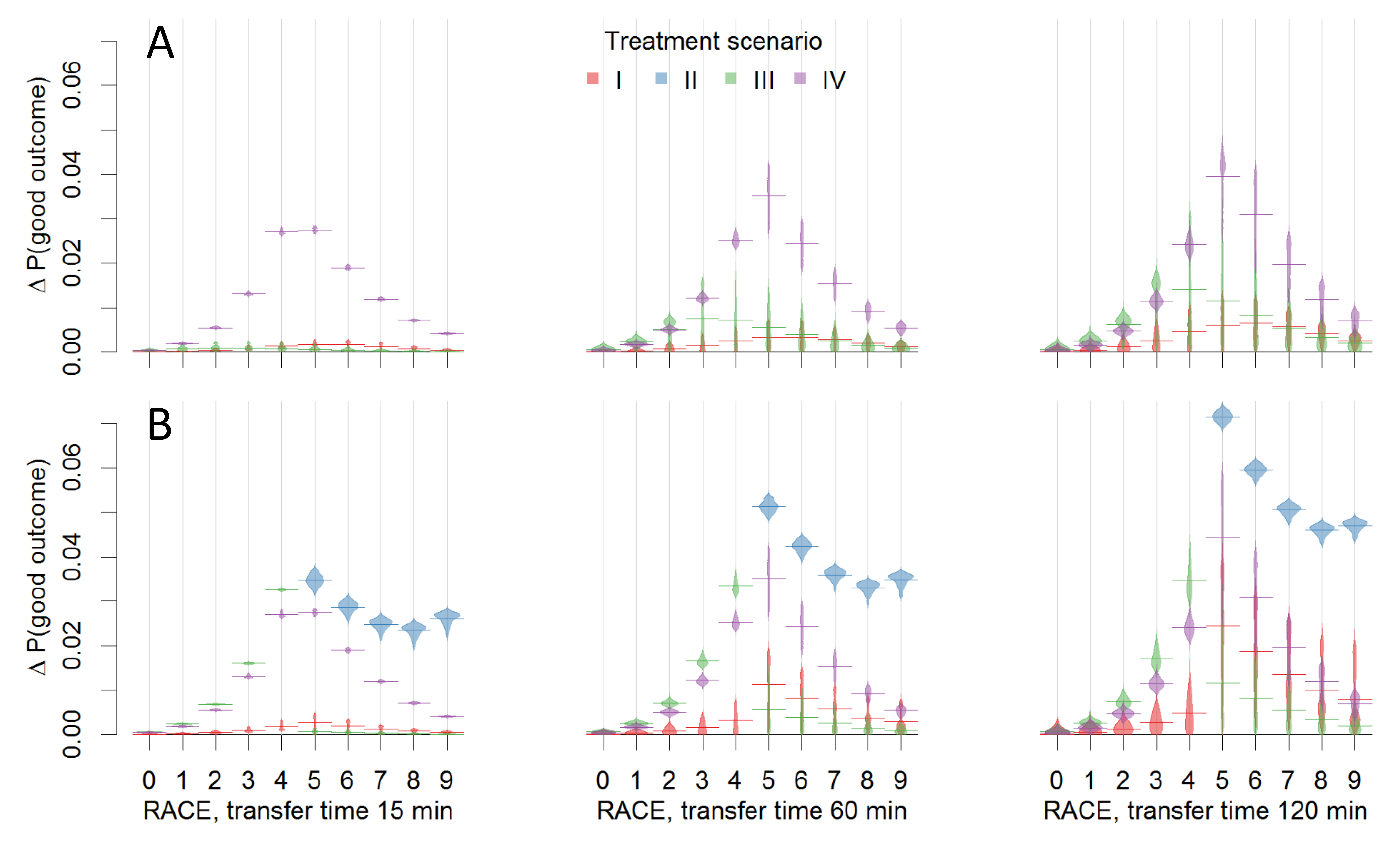

Supplement: Supplementary file 1 — Incremental benefit of a hypothetical perfect large vessel occlusion detection scale according to treatment scenario I – IV. Panel A: Displayed are distributions of point-wise differences between the estimated probabilities of good outcome (modified Rankin scale score ≤2) associated with a prehospital triage strategy based on a hypothetical perfect large vessel occlusion (LVO) detection tool and the estimated probabilities of good outcome associated with a prehospital triage strategy based on optimal rapid arterial occlusion evaluation (RACE) scale cutoff scores, for different stroke severities and different transfer time settings (left to right: 15, 60, and 120 min). Panel B: Displayed are distributions of point-wise differences between the estimated probabilities of good outcome (modified Rankin scale score ≤2) associated with a prehospital triage strategy based on a hypothetical perfect LVO detection tool and the estimated probabilities of good outcome associated with a prehospital triage strategy based on a RACE cutoff score ≥ 5, for different stroke severities and different transfer time settings (left to right: 15, 60, and 120 min). Colors represent different treatment scenarios, with the possibility for treatment options defined by standard treatment time windows: symptom onset-tothrombolysis – 270 min; symptom onset-to-groin puncture – 360 min): I, both thrombolysis at the comprehensive stroke center (CSC) under the mothership approach and secondary transfer for endovascular therapy (EVT) under the drip and ship approach possible. II, thrombolysis at the CSC under the mothership approach not possible, secondary transfer for EVT under the drip and ship approach possible. III, thrombolysis at the CSC under the mothership approach possible, secondary transfer for EVT under the drip and ship approach not possible. IV, neither thrombolysis at the CSC under the mothership approach nor secondary transfer for EVT under the drip and ship approach possible. Horizontal [file 12883_2018_1021_MOESM1_ESM.tif]

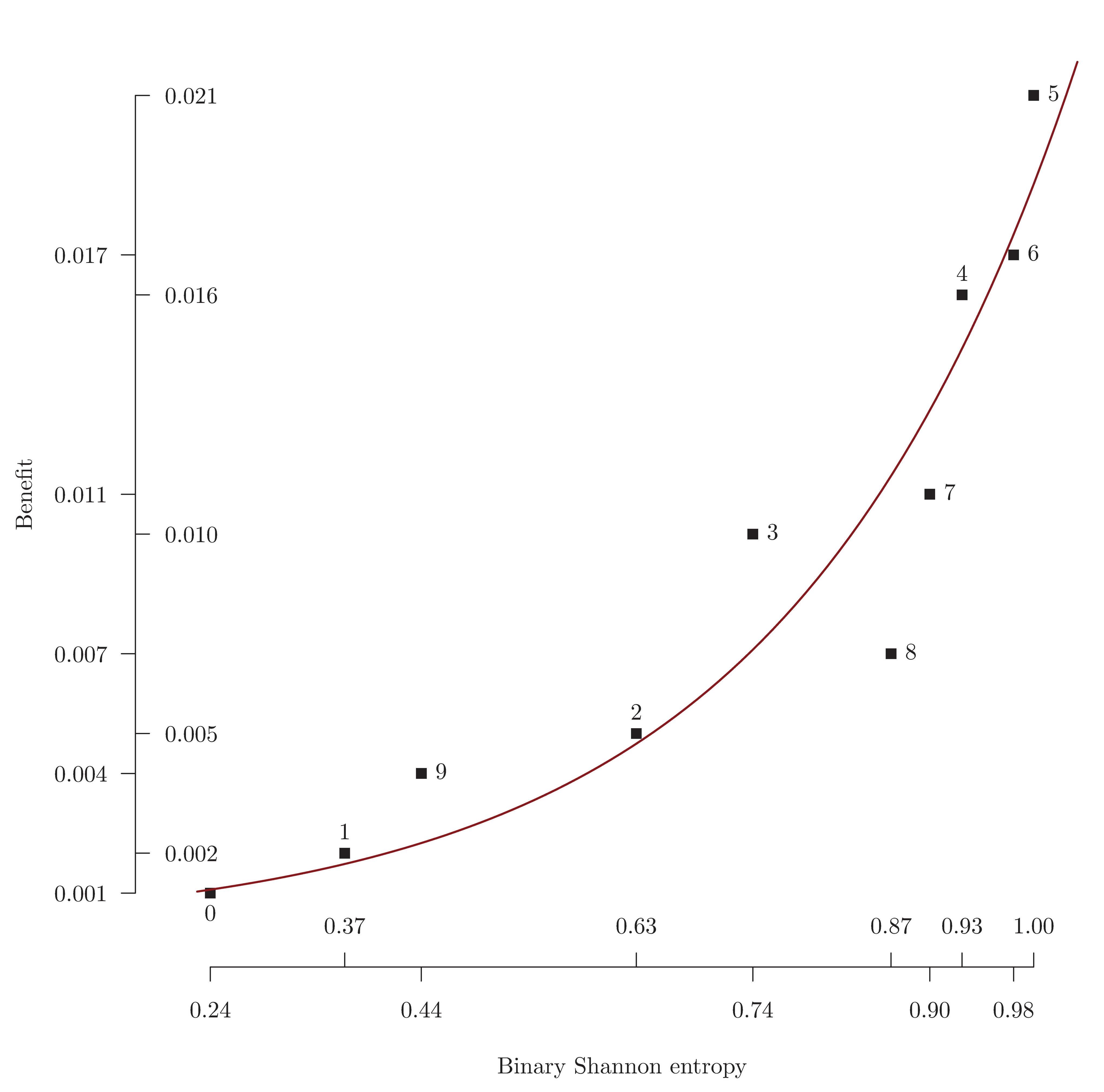

Supplement: Supplementary file 2 — Relationship between the uncertainty with regards to the presence of large vessel occlusion and the incremental benefit of a hypothetical perfect large vessel occlusion detection tool over optimal rapid arterial occlusion evaluation scale cutoff scores. Uncertainty with regards to the presence of large vessel occlusion (LVO) among patients with ischemic stroke in each rapid arterial occlusion evaluation (RACE) scale score category is quantified using the Shannon entropy -p x log2 p - (1-p) x log2 (1-p). Here, p denotes the probability of the presence of LVO among patients with ischemic stroke in a given RACE score category. Incremental benefits of a hypothetical perfect LVO tool correspond to average values in a geographic environment with a transfer time between PSC and CSC of 120 minutes. Labels indicate RACE score categories. (TIFF 1292 kb) [file 12883_2018_1021_MOESM2_ESM.tif]
